# BRAF抑制剂治疗非小细胞肺癌的进展

**DOI:** 10.3779/j.issn.1009-3419.2016.10.13

**Published:** 2016-10-20

**Authors:** 晓艳 康, 楠 朱, 霞 宋

**Affiliations:** 1 030013 太原，山西医科大学附属肿瘤医院呼吸科 Department of Respiratory, Shanxi Cancer Hospital, Affiliated Cancer Hospital of Shanxi Medical University, Taiyuan 030013, China; 2 030012 太原，山西省人民医院消化科 Department of Gastroenterology, Shanxi Province People's Hospital, Taiyuan 030012, China

**Keywords:** 肺肿瘤, BRF, 抑制剂, Lung neoplasms, BRF, Inhibitors

## Abstract

近年来，靶向药物在非小细胞肺癌（non-small cell lung cancer, NSCLC）的治疗中占据着举足轻重的地位，针对表皮生长因子受体（epidermal growth factor receptor, EGFR）的靶向药物已在临床上广泛应用，具有里程碑意义。BRAF抑制剂是针对鼠类肉瘤病毒癌基因同源物B1（V-raf murine sarcoma viral oncogene homolog B1, *BRAF*）基因突变为靶点的靶向药物，对特定优势人群有明显的临床疗效，且毒副作用小，患者易耐受。近期发现，与其他靶向药物一样，BRAF抑制剂也存在耐药现象，其耐药机制正在研究中。本文就BRAF抑制剂的作用机制、临床应用、不良反应及耐药问题进行综述。

## Abstract (en)

In recent years, targeted drugs occupy a pivotal position in the treatment of non-small cell lung cancer (NSCLC), drugs targeting epidermal growth factor receptor (EGFR) has been widely used in clinical practice, it is of milestone significance. V-raf murine sarcoma viral oncogene homolog B1 (BRAF) inhibitors targeted at *BRAF* gene have obviously clinical efficacy to specific advantages populations with little side-effect, and be well tolerated. It is discovered recently that drug resistance also exists in BRAF inhibitors like other targeted drugs, the mechanism of drug resistance is being studied. In this paper, a review were performed in the mechanism, clinical application, adverse reactions and the drug resistance of BRAF inhibitors.

肺癌是全球最常见的恶性肿瘤之一，其发病率及死亡率居高不下，具有预后差，中位生存期短的特点，5年生存率仅17%。肺癌中约85%为非小细胞肺癌（non-small cell lung cancer, NSCLC），传统化疗对该类患者的疗效已达平台期^[1]^，近年来以表皮生长因子受体（epidermal growth factor receptor, EGFR）为靶点的靶向治疗成为人们关注的焦点，随着新的驱动基因不断被发现，靶向药物在NSCLC的治疗中占据着不可忽视的作用。鼠类肉瘤病毒癌基因同源物B1（V-raf murine sarcoma viral oncogene homolog B1, BRAF）是NSCLC的一个驱动基因，其突变率约为0.5%-4.0%，多见于腺癌、女性患者，大多数为V600E突变类型^[2, 3]^。BRAF抑制剂分为广谱性和选择性两类，临床研究已证实选择性抑制剂对NSCLC有很好的疗效，本文重点综述该类抑制剂在NSCLC中的研究进展。

## 1 BRAF抑制剂的作用机制

肿瘤的发生发展常与信号转导通路的异常激活有关，研究较透彻的是RAS/RAF/MEK/细胞外信号调节激酶（extracellular signal regulated kinase, ERK）通路。一般情况下，该通路接受外界信号刺激后，RAS被激活，作用于RAF，继而活化MEK、ERK，使核内的转录因子磷酸化，从而调控细胞的生长、增殖及凋亡。BRAF属于RAF家族，其突变主要集中在第11和第15外显子，约50%发生于第15外显子上第600位的谷氨酸突变为缬氨酸，两类突变均可使BRAF活性增高，而*BRAF* V600E突变能够模拟T598和S601两个位点的磷酸化作用，最大限度地异常增强BRAF活性，且不依赖于上游RAS激酶的激活，促使RAS/RAF/MEK/ERK通路过度激活，导致肿瘤的产生及侵袭转移^[4, 5]^。BRAF选择性抑制剂可以特异性抑制V600E突变细胞的ERK磷酸化，阻断其下游通路的信号转导，诱导细胞周期停滞及细胞凋亡，抑制肿瘤细胞增殖，具有良好的抗肿瘤活性^[6]^。

## 2 BRAF抑制剂在NSCLC患者中的应用

### 2.1 维罗非尼（Vemurafenib）

Hyman等^[7]^进行了一项维罗非尼治疗BRAF V600E阳性肿瘤的前瞻性研究，在NSCLC队列中，19例患者的客观缓解率（objective response rate, ORR）为42%（95%CI: 20%-67%），无进展生存期（progression-free survival, PFS）为7.3个月，中位总生存期（overall survival, OS）尚未达到，但初步的年总生存率达66%，显示了维罗非尼的疗效。2015年欧洲肺癌大会上Gautschi教授^[8]^公布了回顾性EURAF队列研究的结果，研究收集了35例含有BRAF突变的晚期肺腺癌患者，在2012年-2014年接受BRAF抑制剂治疗，其中29例接受维罗非尼，9例接受达拉非尼，患者总缓解率为53%（95%CI: 35.1%-70.2%），中位PFS和OS分别为5个月和10.8个月。维罗非尼组可达到96%的疾病控制率（disease control rate, DCR），表明含有*BRAF*突变的肺腺癌患者使用BRAF抑制剂可以获益。一项法国的Ⅱ期临床试验（NCT02304809）正在招募中，旨在评估维罗非尼单药用于BRAF V600E阳性的实体瘤的安全性及疗效。

Gautschi等^[9]^于2012年报道了1例BRAF V600E阳性的肺腺癌患者，应用维罗非尼2周后复查影像学、细胞学及病理学均显示病灶对维罗非尼敏感，这是首例维罗非尼治疗肺腺癌有效的病例。有关维罗非尼治疗肺腺癌脑转移也有报道^[10]^：患者含有*BRAF* V600E突变，应用维罗非尼4周后脑转移灶疗效评价达部分缓解（partial response, PR），2个月后维持稳定（stable disease, SD），缓解期可达4个月，表明维罗非尼能够通过血脑屏障来发挥疗效。

### 2.2 达拉非尼（Dabrafenib）

达拉非尼的Ⅰ期临床试验^[11]^显示其良好的安全性和耐受性。一项单臂多中心开放标签的Ⅱ期临床研究的中期结果显示，达拉非尼用于经治的*BRAF*V600E突变的NSCLC患者达到了32%的ORR（95%CI: 22%-44%）和56%的DCR（95%CI: 45%-68%），基于此结果，食品药品监督管理局（Food and Drug Administration, FDA）授予了达拉非尼突破性疗法认定。近期，该项临床研究的最终结果被发表在柳叶刀杂志^[12]^，78例经治的患者中，26例出现应答，总缓解率为33%（95%CI: 23%-45%），中位PFS为5.5个月（95%CI: 2.8-6.9），中位OS为12.7个月（95%CI: 7.3-16.9）。6例初治患者有4例达到PR。证实达拉非尼对*BRAF* V600E突变的NSCLC患者有良好的抗肿瘤活性，可作为肺癌人群的一个治疗选择。

### 2.3 其他BRAF选择性抑制剂

除达拉非尼和维罗非尼研究较多外，其他BRAF选择性抑制剂还在临床试验中。Encorafenib（LGX818）和MEK162联合治疗*BRAF* V600E突变的实体瘤的Ⅰ期剂量爬坡试验显示了较好的安全性^[13]^，其Ⅱ期试验正在招募中（NCT01543698）。BGB-283目前正在进行多中心、开放标签、剂量爬坡的Ⅰ期临床试验（NCT02610361），主要评价最大耐受剂量、药动学、药效学以及初步的抗肿瘤活性。

## 3 BRAF抑制剂的不良反应

达拉非尼治疗NSCLC常见的不良反应^[12]^大多属于1级-2级水平，包括皮疹（19%）、皮肤角化（29%）等皮肤毒性，恶心（26%）、呕吐（19%）、腹泻（15%）等胃肠道反应，以及发热（33%）、疲乏（25%）、脱发（21%）等现象，一般情况下患者可以耐受。最严重的不良反应是皮肤鳞状细胞癌（12%），属于3级-4级，但多为高分化肿瘤，局部切除后无复发或转移，达拉非尼也不需要减量或停用。维罗非尼的不良反应^[7]^与达拉非尼相似。整体来讲，与传统化疗产生的毒副作用相比，BRAF抑制剂的不良反应较轻微，患者耐受性良好。

## 4 BRAF抑制剂耐药问题

### 4.1 耐药机制

BRAF抑制剂对BRAF V600E阳性的NSCLC患者疗效肯定，但部分患者会产生获得性耐药。黑色素瘤中BRAF抑制剂的耐药机制研究较为透彻^[14]^，肿瘤细胞主要通过2种途径产生耐药性：一种是通过COT激酶、PDGFRβ过表达及*NRAS*、*MEK*突变重新激活ERK信号通路，另一种是通过激活细胞膜表面酪氨酸激酶受体（RTK），建立不依赖于ERK磷酸化的新的信号传导通路。BRAF抑制剂在NSCLC中的获得性耐药机制尚不明确，1例^[15]^对达拉非尼获得性耐药的NSCLC患者，耐药后再次行基因检测新出现了*KRAS*突变，推测KRAS可能导致了达拉非尼耐药。

有关BRAF抑制剂的原发耐药研究较少，多为个案报道。有研究者^[16]^用三维模型初步说明了*BRAF* G469L突变对维罗非尼固有耐药。1例^[17]^晚期肺腺癌患者，在接受6周期含铂双药化疗及4周期单药维持治疗后疾病进展，肝转移灶穿刺活检提示*BRAF* V600E突变，2014年3月开始口服维罗非尼，6月行正电子发射型计算机断层显像（positron emission computed tomography, PET）/计算机断层扫描（computed tomography, CT）显示疾病进展，提示该治疗失败。体外研究结直肠癌对维罗非尼耐药时推测BRAF抑制剂能够导致EGFR通路代偿活化，当EGFR水平过高，足以抵消BRAF V600E的封闭时，耐药发生。亚洲肺腺癌患者的*EGFR*突变率高达51%^[18]^，提示结直肠癌对BRAF抑制剂耐药的机制在肺腺癌中可能同样适用。

### 4.2 耐药后治疗策略

#### 4.2.1 更换BRAF抑制剂

有文献^[19]^报道BRAF V600E阳性的肺腺癌患者，二线使用维罗非尼4个月后病情进展，换用达拉非尼后症状明显缓解，8周后行PET/CT显示代谢良好，这是首个BRAF V600E阳性的NSCLC患者使用维罗非尼病情进展后对达拉非尼有效的病例报道。Gautschi的EURAF队列研究中^[8]^，3例使用维罗非尼进展后接受达拉非尼治疗的患者，有1例疗效评价达PR。维罗非尼和达拉非尼同属于BRAF选择性抑制剂，维罗非尼耐药后使用达拉非尼有疗效可能是因为两者之间不存在交叉耐药，且部分肿瘤细胞在短暂的治疗间歇期后可以对BRAF抑制剂重新敏感。

#### 4.2.2 联合其他靶向药物治疗

研究表明，MEK抑制剂能够抑制BRAF介导的肿瘤细胞的增殖^[4]^，与BRAF抑制剂联用可以促进肿瘤细胞凋亡，延缓疾病进展^[20]^。在黑色素瘤治疗领域，BRAF抑制剂联用MEK抑制剂比单药治疗更能提高患者的PFS和OS^[21, 22]^。前临床证据显示，肺癌治疗也存在相似现象，维罗非尼联合曲美替尼（MEK抑制剂）比联合厄洛替尼（EGFR抑制剂）更能够协同抑制肿瘤细胞生长，且两药联合的疗效较单药好^[23]^。评价达拉非尼和曲美替尼联用的安全性和疗效的临床试验正在招募中（NCT01336634）。临床前研究^[24]^显示，泛-RAF抑制剂与MEK抑制剂联合可以克服原发性或获得性BRAF抑制剂耐药。

## 5 小结与展望

NSCLC是一个由多因素、多基因经过复杂的信号转导产生的疾病，其特点决定了NSCLC的治疗不能只依靠单一的药物，应该针对基因突变及人群特点进行个体化治疗。BRAF抑制剂对BRAF突变的NSCLC患者有很好的临床疗效，能够进行针对性的治疗，而且研究发现，BRAF突变有可能是EGFR抑制剂耐药的原因^[25, 26]^，相关机制正在研究中，同时也有许多问题有待解决：①*BRAF*突变的检测未列为常规，导致遗漏一部分可能受益的患者；②存在如*EGFR*等高突变频率的患者，如何将BRAF抑制剂与其他靶向药物进行整合治疗；③明确BRAF抑制剂原发及继发耐药的机制。随着这些问题的解决，相信BRAF抑制剂能够有更广阔的应用前景，使NSCLC患者在个体化治疗中更多地获益。
